# Taste receptors, innate immunity and longevity: the case of TAS2R16 gene

**DOI:** 10.1186/s12979-019-0146-y

**Published:** 2019-02-23

**Authors:** Alberto Malovini, Giulia Accardi, Anna Aiello, Riccardo Bellazzi, Giuseppina Candore, Calogero Caruso, Mattia Emanuela Ligotti, Anna Maciag, Francesco Villa, Annibale A. Puca

**Affiliations:** 1 Laboratory of Informatics and Systems Engineering for Clinical Research, Istituti Clinici Scientifici Maugeri, 27100 Pavia, Italy; 2 Section of General Pathology, Department of Biomedicine, Neurosciences and Advanced Technologies, University of Palermo, Corso Tukory 211, 90134 Palermo, Italy; 3 Department of Electrical, Computer and Biomedical Engineering, University of Pavia, 27100 Pavia, Italy; 4 Cardiovascular Research Unit, IRCCS MultiMedica, 20138 Milan, Italy; 5 Department of Medicine, Surgery and Dentistry “Scuola Medica Salernitana”, University of Salerno, 84081 Baronissi, SA Italy

**Keywords:** Bitter taste receptors, Case control study, GWAS, Innate immunity, Longevity, TAS2R16 gene

## Abstract

**Background:**

Innate immunity utilizes components of sensory signal transduction such as bitter and sweet taste receptors. In fact, empirical evidence has shown bitter and sweet taste receptors to be an integral component of antimicrobial immune response in upper respiratory tract infections. Since an efficient immune response plays a key role in the attainment of longevity, it is not surprising that the rs978739 polymorphism of the bitter taste receptor TAS2R16 gene has been shown to be associated with longevity in a population of 941 individuals ranging in age from 20 to 106 years from Calabria (Italy). There are many possible candidate genes for human longevity, however of the many genes tested, only APOE and FOXO3 survived to association in replication studies. So, it is necessary to validate in other studies genes proposed to be associated with longevity. Thus, we analysed the association of the quoted polymorphism in a population of long lived individuals (LLIs) and controls from another Italian population from Cilento.

**Methods:**

The analysis has been performed on data previously obtained with genome-wide association study on a population of LLIs (age range 90–109 years) and young controls (age range 18–45 years) from Cilento (Italy).

**Results:**

Statistical power calculations showed that the analysed cohort represented by 410 LLIs and 553 young controls was sufficiently powered to replicate the association between rs978739 and the longevity phenotype according to the effect size and frequencies described in the previous paper, under a dominant and additive genetic model. However, no evidence of association between rs978739 and the longevity phenotype was observed according to the additive or dominant model.

**Conclusion:**

There are several reasons for the failure of the confirmation of a previous study. However, the differences between the two studies in terms of environment of the population adopted and of the criteria of inclusion have made difficult the replication of the findings.

## Background

Senses can be defined as systems that consist of groups of sensory cells responding to specific physical phenomenon and corresponding to particular brain regions where the received signals are interpreted. In humans, classically, we consider five senses, hearing, smell, sight, taste, touch. Concerning taste sense, humans are able to discriminate five tastes through tongue receptors: sweet, umami, sour, salty, and bitter, which are referred as the basic tastes. Taste perception plays a key role in food preference, dietary habits, and other health issues. Recent research has shown that taste receptors are also expressed in a myriad of other tissues, from the airway and gastrointestinal epithelia to the pancreas and brain, where they participate in a variety of biological processes. Growing evidence suggests that genetic variants of taste receptors play a role in disease aetiology, serving as modifying factors [1, 2].

The immune system and nervous system communicate with one another using a common chemical language. Thus, immune system is considered as our ‘sixth sense’ by informing the nervous system about the presence of pathogens, allergens and cancer cells that the body cannot otherwise hear, see, smell, taste or touch. They communicate via a complete bidirectional circuit sharing both ligands and receptors such as neurotransmitters, neuroendocrine hormones and cytokines [3, 4].

Considering the above statement, it is intriguing that several mechanisms of innate immunity utilize components of sensory signal transduction such as bitter and sweet taste receptors. In fact, empirical evidence has shown bitter and sweet taste receptors to be an integral component of antimicrobial immune response in upper respiratory tract infections. In fact, they have recently been shown to be sentinels of defence against infection in the airway, where they function as a novel arm of innate immunity, as described in detail by Lee and Cohen [5]. Emerging evidence also supports the hypothesis that taste receptors serve similar immune roles in at least some of the other tissues in which they are expressed [6].

Because of the role of an efficient immune response in the attainment of longevity [7, 8], it is not surprising that some polymorphism of the bitter taste receptor genes i.e. taste receptor, type 2, member 16, 4 and 5 (TAS2R16, TAS2R4 and TAS2R5 respectively) has been shown to be associated with longevity in a population of 941 individuals ranging in age from 20 to 106 years from Calabria (Italy). After correction for multiple testing only one single nucleotide polymorphism (SNP), rs978739, showed a statistically significant association with longevity (*p*  =  0.001). In particular, the frequency of homozygotes A/A increased gradually from 35% in the subjects aged 20 to 70 up to 55% in centenarians [9].

Exceptional long living individuals (LLIs), i.e. people belonging to the 5 percentile of the survival curve, are genetically predisposed to reach extreme ages. There are many possible candidate genes for human longevity, however of the many genes tested, only APOE and FOXO3 survived to association in replication studies. So, it is necessary to validate in other studies genes proposed to be associated with longevity [10, 11].

Therefore, in the present paper, to validate this datum, we analysed the association of the previous quoted SNP in a population of LLIs and controls from another Italian population i.e. Southern Italian Centenarian Study (SICS) form Cilento [12, 13].

## Results and discussion

Statistical power calculations showed that the analysed cohort represented by 410 LLIs and 553 young controls [13] was sufficiently powered (1 – β ≥ 0.85) to replicate the association between rs978739 and the longevity phenotype according to the effect size and frequencies described in Campa et al., [9], under a dominant and additive genetic model. The statistical power to replicate the association of rs6466849, rs860170, rs2233998 and rs2227264 was extremely low (1 – β < 0.80) [9] therefore they were not analysed.

The imputed SNP rs978739 (Minor Allele = G with Minor Allele Frequency (MAF) = 0.32) did not significantly deviate from the Hardy Weinberg Equilibrium (HWE) in the control population (*p* = 0.63), was characterized by no missing values (0%) and had an average maximum posterior call of 0.999.

No evidence of association between rs978739 and the longevity phenotype was observed according to the additive (odd ratio (OR) per copy of the G allele = 0.92, 95% CI = 0.76–1.12, *p* = 0.427) or dominant model (OR GG/GA vs. AA = 1.01, 95% CI = 0.78–1.30, *p* – value = 0.955) (Table 1).

**Table 1:** Results from association tests. ID = dbSNP ID; m = minor allele; M = Major allele; MAF = minor allele frequency; Model = genetic model tested; OR (95% CI) = Odds Ratio and 95% Confidence Interval; *p* = *p*-value

ID	m	M	MAF	Model	OR (95% CI)	*P*
rs978739	G	A	0.32	Additive (per copy of G)	0.92 (0.76–1.12)	0.427
				GA vs. AA	1.09 (0.83–1.43)	
				GG vs. AA	0.69 (0.43–1.10)	
				Dominant (GG/GA vs. AA)	1.01 (0.78–1.30)	0.955

Theoretically, there are several reasons for the failure of the confirmation of a previous study [14, 15]. They include the followings. 1) The lack of statistical power either in the discovery or in the replication set. In fact, a result should be supported by an adequate number of study participants, calculated on the number of hypothesis tested and on the frequency of the genetic variant analysed. Indeed, more hypothesises and rare variants require larger populations that one hypothesis and a common variant. If we consider that many studies are unpublished if negative, the threshold of significance should take multiple testing into account even if only one test is published. It is possible that in the previous finding [9], statistics do not consider this multiple testing issue generating a false positive result. 2) The generation of false positives due to the population stratification. In fact, if we compare cases with controls that are not balanced in terms of genetic background, the observed skewing is probably due to the differences in frequency unrelated to the phenotype but depending on the genetic background, the so called genetic admixture. The paradigmatic example is that coming from a study of the association between a GM haplotype and non-insulin-dependent diabetes mellitus on Pima Indians. This study showed an association of the haplotype that disappeared when analysis was restricted to full-heritage Indians [16]. The population adopted by the present study has been controlled for genetic admixture through genetic component analysis and exclusion of outliers [13]. 3) The differences in adopted criteria for inclusion among studies (age of cases and controls, the familiar component of their longevity) and the different environmental factors among analysed populations that could count for different demographic pressures. Indeed, there is an open discussion on the opportunity to replicate the data using populations belonging to the same area. On the other hand, if an association is replicated in heterogeneous populations the results can be generalized to entire community.

## Conclusion

The differences between the two studies in terms of environment of the population adopted and of the criteria of inclusion have made difficult the replication of the findings.

## Methods

The analysis has been performed on data previously obtained with genome-wide association study (GWAS) on a population of LLIs (age range 90–109 years) and young controls (age range 18–45 years). They had been recruited as part of the SICS. The LLIs were thoroughly investigated for demographic characteristics, medical history (past and present diseases), level of independence and cognitive status. All subjects donated blood samples for DNA study and gave written informed consent to the study, which was approved by Ethical Committee of Multimedica Hospital. All methods were performed in accordance with the relevant guidelines and regulations. The study was conducted in accordance with the ethical principles that have their origin in the Declaration of Helsinki.

Genotyping had been carried out with the Infinium II Assay-HumanHap BeadChip 317 K duo system (Illumina, San Diego, California) using standard protocols of the Illumina HumanHap 317 Duo workflow (Illumina, San Diego, CA). All genotypes had been evaluated using a quantitative quality score called GenCall score. The initial screening dataset was represented by 466 LLIs and 624 controls. After quality control, it was looked for evidence of genetic population stratification on a subset of 454 LLIs and 591 young controls. After outliers were removed (*n* = 82), the final GWAS dataset was composed of 963 samples, of which 410 were cases (age range, 90–109 years; male/female ratio, 1.4, and 553 were controls (age range, 18–48 years; male/female ratio, 1.56).

Statistical power calculations were performed by the Quanto software (http://biostats.usc.edu/Quanto.html) based on results described in Campa et al. [9] except for the sample size of the replication cohort [13]. The significance threshold was set to α = 0.05.

Concerning imputation of unobserved genotypes, pre-phasing was performed by the SHAPEIT2 software [17] increasing the number of states up to 500 in order to improve the phasing accuracy and using the reference genetic map for chromosome 7, where TAS2R16 gene is located [9]. The deriving haplotypes were then used as input during the imputation of unobserved genotypes that was performed by the Impute2 software [18].

The SNPTEST software tool was used to test for association between SNP and the longevity phenotype using the score method under the dominant and additive genetic models and to test for statistically significant deviations from the HWE.

## Abbreviations

GWAS: Genome-wide association study
HWE: Hardy Weinberg Equilibrium LLI: Long-living individual
MAF: Minor Allele Frequency SNP: single nucleotide polymorphism
SICS: Southern Italian Centenarian Study TAS2R: taste receptor, type 2
